# Cancer Microenvironment and Inflammation: Role of Hyaluronan

**DOI:** 10.3389/fimmu.2015.00169

**Published:** 2015-04-14

**Authors:** Dragana Nikitovic, Maria Tzardi, Aikaterini Berdiaki, Aristidis Tsatsakis, George N. Tzanakakis

**Affiliations:** ^1^School of Medicine, University of Crete, Heraklion, Greece

**Keywords:** cancer microenvironment, inflammation, hyaluronan, RHAMM, CD44

## Abstract

The role of inflammation in the development of cancer was described as early as the nineteenth century. Abundant evidence supports the preposition that various cancers are triggered by infection and chronic inflammatory disease whereas, evading immune destruction has been proposed as one of the new “hallmarks of cancer.” Changes of the tumor microenvironment have been closely correlated to cancer-mediated inflammation. Hyaluronan (HA), an important extracellular matrices component, has become recognized as an active participant in inflammatory, angiogenic, fibrotic, and cancer promoting processes. This review discusses how HA and specific HA-binding proteins participate in and regulate cancer-related inflammatory processes.

## Cancer Microenvironment

The role of inflammation in the development of cancer was described as early as 1863, by Rudolf Virchow, who hypothesized that cancer arises from inflammatory sites, “lymphoreticular infiltration” (1). In the last decades, Virchow’s postulation has been supported by abundant evidence that various cancers are triggered by infection and chronic inflammatory disease (2). On the other hand, an inflammatory response is also detectable in tumors that are not causally related to inflammation (3). Following cell transformation to a malignant state, the inflammatory mediators are involved in tumor growth, by stimulating the proliferation of tumor cells and by evading immunosurveillance. Notably, the inflammation orchestrated by the tumor is aberrant and promotes the recruitment and/or the induction of cells that, besides having a role in the direct promotion of the tumor progression, are also endowed with immunosuppressive properties. Indeed, evading immune destruction has been proposed as one of the new “hallmarks of cancer” (4).

The consecutive steps of tumor growth, local invasion, intravasation, extravasation, and invasion of anatomically distant sites as well as immunosuppression are obligatorily perpetrated through specific interactions of the tumor cells with their microenvironment (3, 5). Extracellular matrices (ECMs) represent a complex network of proteins and glycosaminoglycans (GAGs), which define the structure of tissues *in vivo* and are critically important for cell growth, survival as well as differentiation, and key to various disease processes including inflammation and cancer (6–10). During cancer progression, significant changes can be observed in the properties of ECM components, which deregulate the behavior of stromal cells, promote tumor-associated angiogenesis and inflammation, and lead to generation of a tumorigenic microenvironment (11–14).

Hyaluronan (HA), an important ECM component, has become recognized as an active participant in inflammatory, angiogenic, fibrotic, and cancer promoting processes. HA and its binding proteins regulate the expression of inflammatory genes, the recruitment of inflammatory cells, the release of inflammatory cytokines and thus, ultimately can attenuate the course of inflammation (15). Surprisingly, HA is a relatively simple molecule being an anionic, non-sulfated GAG in the 5000–20,000,000 Da molecular weight range. It is a polymer of disaccharides composed of alternating *N*-acetylglucosamine (GlcNAc) and glucuronic acid (GlcA) units (16). HA is unique among GAGs because it neither contains sulfate groups nor is it covalently linked with a core protein (17). This GAG is synthesized by three types of integral membrane proteins denominated HA synthases: HAS1, HAS2, and HAS3. The HAS enzymes synthesize different HA sizes by repeatedly adding glucuronic acid and *N*-acetylglucosamine to the nascent polysaccharide while it is extruded through the cell membrane into the extracellular space (18). Specifically, HAS1 and HAS2 produce very high molecular weight HA (HMWHA) up to 2000 kDa (19). The degradation of HA within tissues, on the other hand, is performed by enzymes known as hyaluronidases (HYAL). In humans, there are at least seven types of hyaluronidase-like enzymes with HYAL1 and 2 being the most important. HYAL hydrolyzes the β(1–4) glycoside bond between *N*-acetyl-d-glucosamine and d-glucuronic acid, which results in the production of fragments of different sizes (20). It is noteworthy that the size of HA chains affects its biological functions. Indeed, oligosaccharides that result from HA degradation and low-molecular-weight HA (LMWHA), defined as fragments in the 5–500 kDa range (20) are able to induce the processes of inflammation and angiogenesis. HMWHA (1000–2000 kDa), on the other hand, is present in intact tissues and is antiangiogenic as well as immunosuppressive (21–23).

## Hyaluronan Accumulation and Turnover in Cancer Tissue

The alteration of HA deposition in various malignancies has been well established (13, 24). Thus, a significant number of studies show that HA deposition is elevated in various types of cancer tissues including colon, breast, lung, and prostate cancer (25–27). The magnitude of the HA accumulation both around the tumor cells and in the surrounding stroma strongly correlates with the aggressiveness of cancers by enhancing processes involved in malignant growth, like cell proliferation, invasion, metastasis, and tumor–stroma interactions (13, 24, 28). It is widely accepted that HAS mRNA levels determine the synthesis of HA (29, 30). The mechanisms, however, of HA accumulation vary. Thus, it has been suggested that fibroblast growth factor receptor (FGFR) activation induces accumulation of HA within the ECM, through HAS upregulation (31, 32). Furthermore, abnormal pre-mRNA splicing, leading to intracellular or extracellular HA synthesis by HASs, is suggested to contribute to the initiation and progression of various types of cancer (33). Importantly, an increased HYAL expression has been associated with tumor progression in a number of cancer types (34, 35). It is noteworthy that, tumor tissues are characterized by increased production of reactive oxygen species (ROS) resulting from increased metabolic activity, enhanced activity of NADPH oxidase (NOXs), or mitochondrial dysfunction of tumor tissues (12, 36). GAGs are very susceptible to ROS-induced degradation either via ^•−^OH radical action, which is a product of ONOO^−^ decomposition (37) or through radical ^•^NO action. Importantly, the balance between radical ^•^NO and $O_{2}^{•}$ radical determines which GAG component of the ECM is destroyed and this selective degradation may be important in regulating specific aspects of the disease processes (38, 39). Therefore, on one hand, there is an established upregulation of HA deposition in tumor tissues whereas simultaneous overexpression of HYALs and overproduction of ROS induces HA degradation. Indeed, taking into account HA-size-dependent biological effects, this complex turnover pattern is in fact suggested to confer tumorigenic potential (40). The generation of various HA fragments sizes and their highly specific action on tumor cell functions has been widely established (24, 41–43). The majority of reports up to date indicate that LMWHA fragments support tumor growth and dissemination whereas, HMWHA is suggested to have anti-tumor effects (24, 44, 45). Indeed, excess deposition of HA was found to suppress tumor growth in the absence of HYAL. Thus, the overexpression of HAS in prostate carcinoma cells that are characterized by very low endogenous HA deposition and HAS expression significantly reduces tumor growth kinetics in both the subcutaneous (46, 47) and the orthotopic primary injection site (48). In contrast, results obtained in the fibrosarcoma cell model suggest that HMWHA may be pro-proliferative and enhance motility (49). This may be an unusual property of fibrosarcoma tumors that is opposite to effects observed in tumors that originate within the epithelial compartment. In line with the established pattern of HA effects, the majority of reports suggest that HMWHA protects the integrity of the endothelial barrier. HMWHA was shown to decrease permeability in cancer lymphatic endothelial cell monolayers (45) and actually promote enhancement of vascular integrity, indicative of anti-metastatic effects (44). Opposite effects of LMWHA have been documented (44, 45). A recent report, however, indicates that the augmentation of CXCR4 signaling by HMWHA resulted in increased vessel sprouting and angiogenesis in a variety of assays (50). When interpreting data relevant to HA action, it is important to note that in general HA signaling is cancer type/cell line-specific as HAS3-dependent HA synthesis has been found to suppress cell proliferation by elevating cell cycle inhibitor expression and suppressing G1- to S-phase transition (51) whereas, LMWHA inhibits colorectal carcinoma growth by decreasing tumor cell proliferation and stimulating immune response (42).

## Immunological Aspects of HA in Cancer Progression

Intriguingly, a recently proposed driver model for the initiation and early development of solid cancers associated with inflammation-induced chronic hypoxia and ROS accumulation focuses on HA action. Namely, inflammation-induced chronic hypoxia can ultimately result in the production and export of HA, which will be degraded into fragments of various sizes, serving as tissue-repair signals, which lead to the initial proliferation of the underlying cells (52). In addition, HA degradation products have the ability to induce specific gene expression programs for proteases and cytokines that are necessary for inflammation and matrix remodeling. Several studies have shown that HA fragments activate innate immune responses by interacting with TLR2 and TLR4 and inducing inflammatory gene expression in a variety of immune cells (53–55). There appear to be a feedback regulation here as, proinflammatory cytokines induce HA synthesis and monocyte adhesion in human endothelial cells through HAS2 and the nuclear factor κ-B (NF-κB) pathway (56). As regarding tumor cells, exposure of human melanoma cells to HA fragments leads, via TLR4, to NF-κB activation followed by enhanced expression of matrix metalloprotease (MMP) 2 and interleukin (IL)-8, factors that can contribute to melanoma progression (57). In a recent study, LMWHA (but not HMWHA) was found to preferentially stimulate a physical association between CD44 and TLRs followed by a concomitant recruitment of AFAP-110 and MyD88 into receptor-containing complexes in breast tumor cells. This results in MyD88/NF-κB nuclear translocation, NF-κB-specific transcription, and target gene IL-1β and IL-8 expression. Therefore, LMWHA signaling events lead to proinflammatory cytokine/chemokine production in the breast tumor cells (58). Another example of contrasting LMWHA and HMWHA effects is illustrated by a study performed on human SW-1353 chondrosarcoma cells. HMWHA antagonized the effects of IL-1β by increasing PPARγ and decreasing cyclooxygenase (COX)-2, MMP-1, and MMP-13 levels. Furthermore, in this model, HMWHA promoted Akt, but suppressed mitogen-activated protein kinases (MAPKs) and NF-κB signaling, indicating anti-inflammatory effects. In contrast, chondrosarcoma cells had overall stimulatory responses to oligo-HA as regarding inflammatory genes (59). Inflammation establishes a tissue microenvironment, which tolerates tumor growth and metastasis by setting immunosuppressive mechanisms (60). Therefore, inflammation not only induces carcinogenesis but also makes immune cells incapable of destroying tumor cells (61). It is indicative that LMWHA fragments are able, in a TLR4/IFN-β-dependent pathway, to accelerate the elimination of inflammatory neutrophils by promoting their apoptosis (62). Moreover, in the tumor microenvironment, HA fragments can reprogram neutrophil action. Thus, tumor cell-derived HA fragments through TLR4/PI3K signaling induce early activation and longevity of tumor neutrophils, which in turn stimulate the motility of malignant cells. This skewed inflammatory mechanism represents an example of the positive regulatory loop between tumors and their stroma during neoplastic progression (63). On the other hand, LMWHA treated dendritic cells increased IFN-γ production, and secreted lower levels of the immunosuppressive IL-10 coupled with higher proliferation rates and increased motility. Moreover, these preconditioned dendritic cells elicited induced immunity in a murine colorectal cancer model (42).

Recently, the importance of HA-coated extracellular vesicles in carcinogenesis has been suggested. HA is suggested to be carried on the surface of these vesicles in tissues and body fluids, creating beneficial environments by itself, or by associated molecules, for the invasion and metastasis of cancer cells (52, 64). HA transferred by these vesicles could putatively contribute to cancer-related inflammatory processes.

## HA Receptors in Inflammation

Biological functions of HA are mediated by its molecular interaction with HA-binding proteins, called hyaladherins, and as a result, gain new biological identities (65, 66). In more detail, HA binds to its specific cell-surface receptors, including CD44, receptor for hyaluronan-mediated motility (RHAMM), and intercellular adhesion molecule 1 (ICAM-1), activating the transduction of a wide range of intracellular signals (67, 68). These HA receptor interactions are implicated in both physiological and pathological conditions, as they regulate cellular processes such as morphogenesis, wound healing, and inflammation (68–70). CD44 is a cell-surface glycoprotein encoded by a single gene, although there are a number of isoforms expressed in a number of cells and tissues as a result of alternative splicing (71). Importantly, it has been shown that some splice variants such as CD44v-9 and CD44v-6 are involved in tumor metastasis (72, 73) even though CD44 expression pattern cannot always be correlated with malignant progression (74). Moreover, inflammation and malignancy are often associated with sequential proteolytic cleavage of CD44 resulting in a soluble extracellular part of CD44 that most likely regulates cell migration, and to a CD44 intracellular domain that translocates to the nucleus and promotes transcription of different genes including the CD44 gene itself (75, 76). Noteworthy, at sites of inflammation, a concomitant increase in HA synthesis and release of inflammatory mediators can increase the binding avidity of CD44 for HA. Post-translational modifications of CD44 have also been implicated in the transition of an “inactive” low affinity state to an “active” high affinity state of the CD44–HA binding capacity. Other molecules, also produced in the inflammatory milieu, including IL-2, tumor necrosis factor (TNF), and chemokines including MIP-1β, IL-8, and RANTES, can stabilize and increase HA–CD44 interactions (77, 78). Another inflammatory marker, SHAP protein that corresponds to the heavy chains of inter-alpha-trypsin inhibitor family molecules circulating in blood, also stabilizes HA–CD44 interactions (79). Moreover, recently it was suggested that HAS1-dependent HA coat is induced by inflammatory agents and glycemic stress, mediated by altered presentation of either CD44 or HA and can offer a rapid cellular response to injury and inflammation (80). Such interactions are important for the regulation of CD44-mediated leukocyte migration to sites of inflammation (78, 81) as well as monocyte/macrophage retention and activation in inflammatory sites (82). Moreover, an alternate immune evasion mechanism, based on the interaction between CD44 on lung cancer cells and extracellular HA has been proposed. In this study, CD44/HA interactions, which reduce both Fas expression and Fas-mediated apoptosis of the cells, result in decreased susceptibility of the cells to T lymphocytes-mediated cytotoxicity through Fas–FasL pathway (83). On the other hand, engagement of CD44 was found to upregulate Fas ligand expression on T cells leading to their activation-induced cell death (84). The versatility of HA/CD44 interactions are illustrated by HA-mediated CD44 interaction with RhoGEF and Rho kinase, which promotes Grb2-associated binder-1 phosphorylation and phosphatidylinositol 3-kinase signaling leading to cytokine (macrophage-colony stimulating factor) production and breast tumor progression (85). There appears to be a backfeed interaction between inflammatory mediators and CD44 expression as, tumor necrosis factor-alpha (TNF-alpha), a major inflammatory cytokine, abundant in the ovarian cancer microenvironment was found to differentially modulate CD44 expression in ovarian cancer cells (86). Some reports, however, propose that CD44 negatively regulates *in vivo* inflammation mediated by TLRs via NF-κB activation, which ultimately leads to proinflammatory cytokine production (87).

Receptor for hyaluronan-mediated motility has been suggested to contribute to “cancerization” of the tumor microenvironment through its wound repair functions including inflammatory cues (69, 88). It is hypothesized that RHAMM could be a member of the damage-associated molecular-pattern (DAMP) molecules, which function as proinflammatory signals (89). This is corroborated by data showing that RHAMM expression was strongly positively correlated to severe infection in immune atopic diseases (90). Moreover, central to the inflammation process, macrophage chemotaxis was found to be upregulated in a RHAMM- and HA-dependent manner (91). Indeed, upon utilization of a RHAMM mimetic peptide, which specifically blocks HA signaling a strong reduction of inflammation and fibrogenesis in excisional skin wounds was determined (69). Furthermore, RHAMM has been identified as an immunologically relevant antigen, strongly expressed in several hematologic malignancies, and associated with both cellular and humoral immunity (92, 93). Indeed, persistent RHAMM expression and decreasing CD8+ T-cell responses to RHAMM in the framework of allogeneic stem cell transplantation or chemotherapy alone might indicate the immune escape of leukemia cells (94). These aspects of RHAMM/HA signaling can be utilized as targets of novel cell-based strategies in cancer. Thus, vaccination with a highly immunogenic peptide, which was derived from RHAMM and, respectively, denominated R3, has demonstrated positive and safe effects in generating CD8+ cytotoxic cellular responses and anti-tumor traits in patients with myelodysplastic syndrome, acute myeloid leukemia, multiple myeloma, as well as chronic lymphocytic leukemia (95, 96).

**Figure 1:**
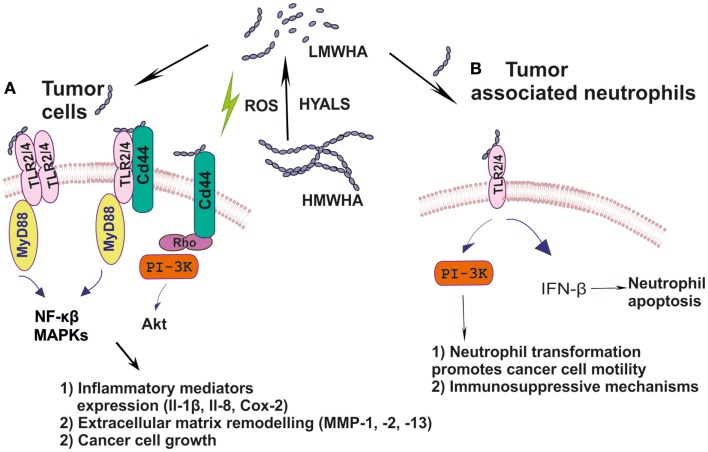
**(A)** Ha fragments through specific interactions with hyaladherins activate intracellular pathways including MAPKs, NF-κβ, and PI3K/Akt, which support tumor cell growth; **(B)** HA by modulating TLR2/4 downstream signaling reprograms inflammatory cells to create a tumor-permissive environment.

ICAM-1 is of great importance for immune response, inflammation, and wound healing. Indeed, it is a key molecule for leukocyte adherence and transendothelial migration with significant HA participation in this regulation (68, 97). ICAM-1/HA interactions have been implicated in various inflammatory processes. Thus, a reduction of ICAM-1 expression, mediated by HA may have an anti-inflammatory role in a rat model of severe non-bacterial cystitis (98). Indeed, the anti-inflammatory effect of HMWHA is suggested to be perpetrated through interactions with more than one hyaladherin, including ICAM-1 (99). These authors show that the inhibition of inflammation promoting cathepsin K and MMP-1 activities is accomplished through joint TLR4, CD44, and ICAM-1 actions. Up to date, however, ICAM-1/HA interactions have not been examined in cancer-induced inflammation. The cancer inflammation-related effects of HA and its respective receptors are schematically depicted in Figure 1.

## Conclusion and Perspectives

The tumor microenvironment plays a key role in cancer progression. Specifically, HA-rich tumor microenvironments regulate important host–tumor interactions and have significant impact on cancer-related inflammatory processes. One area, which holds promise for cancer immunotherapy, is the manipulation of immune responses, ultimately providing a therapy that might “switch” back on the immune system to target the tumor cell. Therefore, by determining the mechanisms through which inflammatory HA fragments are generated in cancer and the respective role of HA receptors will enable us to understand better the contribution of inflammation in malignant disease and perhaps reveal new therapeutic strategies.

## Conflict of Interest Statement

The authors declare that the research was conducted in the absence of any commercial or financial relationships that could be construed as a potential conflict of interest.

## Abbreviations

DAMP, damage-associated molecular-pattern; ECMs, extracellular matrices; GAGs, glycosaminoglycans; HA, hyaluronan; HAS, hyaluronan synthases; HMWHA, high molecular weight HA; LMWHA, low molecular weight hyaluronan; HYAL, hyaluronidases; RHAMM, receptor for hyaluronan-mediated motility; ICAM-1, intercellular adhesion molecule 1; MAPKs, mitogen-activated protein kinases; MMP, matrix metalloprotease; TNF, tumor necrosis factor; TNF-alpha, tumor necrosis factor-alpha.
